# Splenocytes and thymocytes migration patterns between lymphoid organs in pregnancy

**DOI:** 10.1016/j.bbrep.2024.101769

**Published:** 2024-07-02

**Authors:** Gabriela T. Cruz-Cureño, Marina Ch Rosales-Tarteaut, Lourdes A. Arriaga-Pizano, Luvia E. Sánchez-Torres, Denisse Castro-Eguiluz, Jessica L. Prieto-Chávez, Rodolfo Pastelin-Palacios, Ana Flisser, Arturo Cérbulo-Vázquez

**Affiliations:** a Escuela Nacional de Ciencias Biológicas, Instituto Politécnico Nacional, Prolongación Carpio s/n esq, Plan de Ayala, Plutarco Elías Calles, CP 11340, Ciudad de México, Mexico; b Unidad de Investigación Médica en Inmunoquímica, Centro de Instrumentos, Hospital de Especialidades, CMN Siglo XXI, Av. Cuahutemoc 330, Doctores, CP 06725, Ciudad de México, Mexico; c Centro de Instrumentos, Hospital de Especialidades, CMN Siglo XXI, Ciudad de México, Mexico; d Investigador por México, CONAHCyT-Departamento de Investigación Clínica, Instituto Nacional de Cancerología, Ciudad de México, Mexico; e Facultad de Química, Universidad Nacional Autónoma de México, Ciudad Universitaria, Circuito interior, Av. Universidad 3000, CP 04510, Ciudad de México, Mexico; f Departamento de Microbiología y Parasitología, Facultad de Medicina, Universidad Nacional Autónoma de México, Ciudad Universitaria, Circuito interior, Av. Universidad 3000, CP 04510, Ciudad de México, Mexico; g Hospital General de México “Dr. Eduardo Liceaga”. Medicina Genómica, Dr. Balmis 148. Doctores, Alcaldía Cuauhtémoc, CP 06726, Ciudad de México, Mexico

**Keywords:** Cell trafficking, Lymphoid organ, Pregnancy, Flow cytometry

## Abstract

**Background:**

Cell migration is essential for the immune system and is frequently analyzed in adult non-pregnant animals but poorly explored in pregnant animals. However, a physiologic increased size in the spleen and periaortic lymph nodes had been reported in pregnant mice.

**Methods:**

Using a mouse model, we transferred PKH26-stained thymocytes and splenocytes from pregnant or non-pregnant animals to receptor mice in the presence or absence of pregnancy. Percentage of PKH-26 cells and Mean Fluorescence Intensity were calculated. Non-parametric ANOVA analysis was performed.

**Results:**

We detected that the percentage of PKH26+ thymocytes in the spleen, lymph nodes, and peripheral blood is higher in females than in males (p = 0.039). Our results showed a similar frequency of thymocytes and splenocytes from pregnant and non-pregnant mice located in receptor lymphoid organs (p > 0.05). Also, the location of marked cells was similar during the perinatal period (p > 0.05).

**Conclusions:**

The mobility of thymocytes and splenocytes in pregnant and non-pregnant mice is similar. Therefore, we suggest that the larger size of the spleen and periaortic lymph nodes noted previously in pregnant mice could result from the retention of leukocytes in the secondary lymphoid organs.

## Highlights

• Transfer cells are more retained in pregnant mice than in male mice.
• Despite pregnancy, transfer cells are similar in lymphoid tissue of receptors.
• Thymocytes and splenocytes show similar migration with or without pregnancy.

## 1 Background

Cell trafficking is an essential function of the immune system. Cell migration patterns had been recognized for leukocytes egressing from bone marrow and thymus (primary lymphoid tissues) into the blood and afterward to lymph nodes and spleen (secondary lymphoid organs, SLO) [1,2]. Also, thymocyte migration within the thymus has been detected as critical for positive and negative selection [3]. At the same time, naïve T and B cells that egress from the thymus or bone marrow could recognize antigens in the SLO and then proceed to activation and proliferation [4]. Depending on antigen encounter, an alternative behavior is for naïve lymphocytes to egress from SLO, return to the blood through the thoracic duct, and recirculate [5]. In addition, memory cells follow a similar migration pattern as naïve cells; in contrast, effector cells home to non-lymphoid tissue and express their function [6,7].

Also, time is essential to analyze the pattern of cell migration. Mature T cells that egress from the thymus spend minutes in the blood [8,9] and then arrive at the SLO [4,10]. Lymphocytes remain in the SLO for 8–12 h (T cells) or 24 h (B cells) searching for antigen; if they encounter their antigen, they remain in the SLO, become activated, and proliferate; if no antigen is encountered, they leave the SLO and circulate in the blood [8]. The pattern of cell migration also changes throughout the day, and it has been reported that the circadian clock controls leukocyte trafficking [11], where the peak of leukocyte circulation in rodents is during the day [12].

Immune cells' migration patterns haven't been characterized in pregnancy, where the immune system expresses a highly regulated state of immunotolerance. Reports have described increased leukocyte migration to the paraaortic lymph nodes in pregnant mice, with hypertrophy between 14 and 16 days [13]. Also, a reduction in the size of the thymus and an increase in the size of the spleen have been reported previously in pregnancy [14]. These changes are observed in allogeneic and syngeneic systems [[15], [16], [17], [18]]. Like the spleen, the proportion and the absolute number of mononuclear cells in peripheral blood increase during pregnancy [19].

In this study, we aimed to analyze whether cell migration to lymphoid organs depends entirely on the cell or if the pregnancy environment conditions it. We used a stained cell transfer model in mice. Thymocytes or splenocytes from pregnant or non-pregnant animals were isolated, marked, and transferred to pregnant or non-pregnant pairs. We report the proportion and absolute numbers of cells in the thymus, spleen, lymph nodes, and blood. Female mice showed a greater proportion of marked cells in lymphoid organs than male mice. The proportion of splenocytes detected in the spleen was higher than the thymocytes detected in the spleen. Our results suggest the cell condition by itself supports a differential location to the lymphoid organs. This indicates that the migration program is expressed in leukocytes and minimally affected by the pregnant environment.

## 2 Methods

An experimental, prospective, and analytical study was conducted to describe if the frequency of marked leucocytes in lymphoid organs is similar in the absence and presence of pregnancy.

### 2.1 Mice

Female and male BALB/c mice (3 mice per experimental group, we did a convenience sample and expect a minimal variance), 6–8 weeks of age, were obtained from the Bioterio Animal Production and Experimentation Unit (UPEAL-B), UAM-Xochimilco. The biological samples were managed according to the Biosafety Manuals of the Faculty of Medicine of the UNAM. Mice were kept in acrylic boxes (19x29 × 12cm) under constant temperature conditions (23 °C) with standard 12 h light/dark intervals. Food and water were supplied on demand. The hormonal cycle was tested to analyze homogeneous groups of mice, and animals in the estrous cycle were used. Briefly, the vaginal sample was taken every 24 h (four consecutive days) and stained with 0.1 % crystal violet. The colpocytological exams were observed under an optical microscope, showing a physiologic cyclicity.

### 2.2 Collection of peripheral blood

Sevoflurane was used as an inhalation anesthetic; the lack of reflexes, relaxation, and regular breathing was checked before starting the procedure. Mice were placed supine, and peripheral blood was extracted from the mice using a cardiac puncture. Briefly, locating the xiphoid bone, a lateral puncture with an angle of 30° was performed, and exerting gentile negative pressure with the syringe (1 mL volume/25G needle), blood emerged. Then, mice were sacrificed by cervical dislocation. The blood was collected in microtainer tubes (BD, catalog. 363706 NJ, USA). Subsequently, using NH_4_Cl solution (150 mM ammonium chloride, 10 mM KHCO3, and 1 mM EDTA), the erythrocyte lysis was performed. The sample was washed with PBS (Sigma-Aldrich, catalog P3813 St. Louis, MO) at 4 °C and centrifuged at 1500 rpm for 10 min. The suspension was decanted, and the cell button was resuspended in 100 μL. Then, using a Neubauer chamber, cell viability was quantified using the trypan blue exclusion method.

### 2.3 Cell suspension from lymphoid organs

Pregnant (P) and non-pregnant (NP) adult mice of 6–8 weeks under anesthetic were sacrificed by cervical dislocation. Then, the thymus, spleen, axillary, and inguinal lymph nodes were dissected. Cell suspensions were obtained using a fine mesh and washed by centrifugation (PBS at 4 °C, at 1200 rpm for 10 min). The Samples were decanted, filtered, and resuspended in a volume of 500 μL. Cell count and viability were evaluated by the trypan blue exclusion method.

### 2.4 PKH-26 mark of cells

Five million cells were marked using the PKH-26 Red Fluorescent Cell Linker Kits (Sigma-Aldrich, catalog PKH26GL St. Louis, MO). Following the manufacture instructions, cells were resuspended in diluent C (Sigma-Aldrich, catalog CGLDIL St. Louis, MO) and mixed with PKH26 ethanolic dye solution (Sigma-Aldrich, catalog P9691 St. Louis, MO) and incubating 5 min at 37 °C. After incubation, the reaction was stopped with PBS (Sigma-Aldrich, catalog P3813 St. Louis, MO), tubes centrifuged, and cells resuspended in 200 μL of PBS. Cell viability was quantified as previously described. Data acquisition and analysis were performed on a FACSCanto® (BD Biosciences) using the Infinicyt™ software (Cytognos Euroflow®) for data analysis. Fig. 2a-d shows the plots for PKH-26+ thymocyte identification in receptor lymphoid organs and peripheral blood, while Fig. 2e-h shows the plots for PKH-26+ splenocytes in receptor organs and blood.

### 2.5 Transfer of cells

Four cell transfer systems were analyzed: **NP→NP**, **NP→P**, **P→NP**, and **P→P**. The tail was clamped with local heat to dilatate the caudal vein. Using insulin syringes with a 13 mm long (1/2″) and 27G thick needle (BD, catalog 326716 NJ, USA), 5x10^6^ of PKH26+ cells in a volume of 200 μL were transferred at 15–16 days post coitus (DPC), or in the estrous phase if mice were non-pregnant. All cell transfers were performed early in the day (between 9:00 and 11:00 h).

### 2.6 Count of PKH26+cells in receptor lymphoid organs

**Fig. 1:**
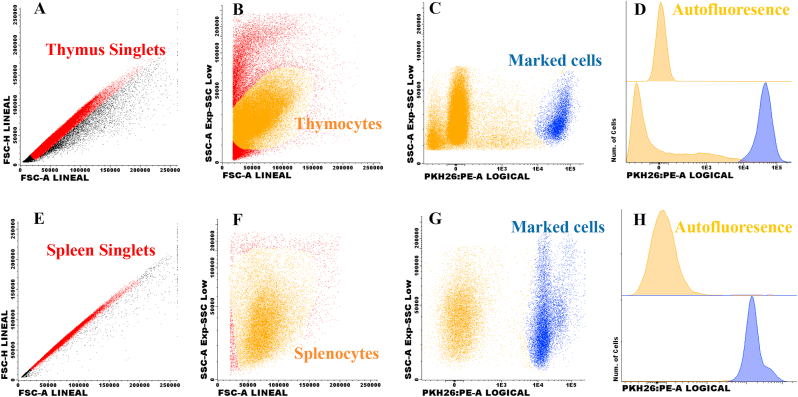
**Thymocytes or splenocytes after labeling with PKH-26**. Cell suspensions were obtained from the thymus or spleen and labeled with PKH-26 as in methods (1A-H). Images show the total cells acquired per organ of non-pregnant mice (n = 3). The top histogram shows unmarked cells, and the bottom histogram shows PKH-26+ in thymocytes (Fig. 1D) and splenocytes (Fig. 1H).

Mice were sacrificed two days after the cell transfer, and lymphoid organs were isolated. One million cells per 100 μL were obtained from the spleen, thymus, lymph node, peripheral blood, or placenta and placed into cytometry tubes (Falcon, catalog 352008 MA, USA). Then, following manufacturer instructions, 300 μL of FACS Lysing Solution was added (BD, catalog 349202 San Jose, CA) and incubated in the dark for 10 min. Then, one mL of PBS was added, and cells were centrifuged at 3000 rpm for 5 min; samples were decanted and resuspended in 100 μL of PBS. Data acquisition was performed on the FACSCanto flow cytometer (BD Biosciences, USA), and Infinicyt cytognos euroflow software was used for analysis (Cytognos SL, Salamanca, Spain). The strategy to identify PHK26+ cells used dot plots graphs (Fig. 1).

**Fig. 2:**
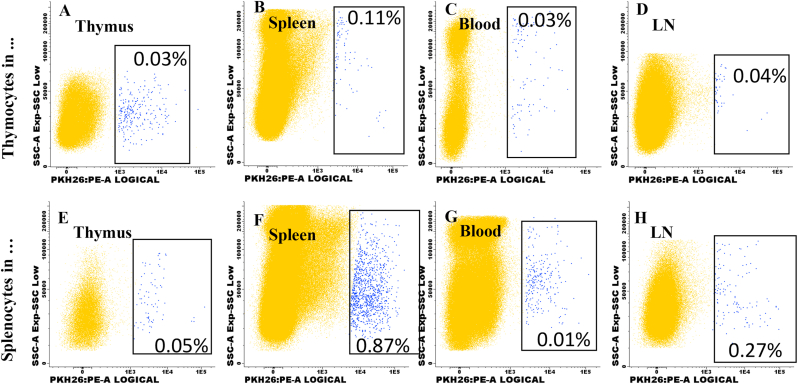
**PKH-26+ thymocytes or splenocytes in receptor organs**. Cell suspensions were obtained from the thymus or spleen and labeled with PKH-26, as in Fig. 1. PKH-26+ cells were identified two days later in receptor lymphoid organs and peripheral blood (2A-H). Images show the total cells acquired per organ of non-pregnant mice (n = 3). Gated cell shows PKH-26+ cells in lymphoid organs and peripheral blood (Fig. 2A–H).

### 2.7 Statistics

Analysis was performed using GraphPad Prism version 6 software (GraphPad Software Inc. La Jolla, California, USA). A non-parametric Kruskal Wallis test with Dunn post-test multiple comparisons test was used to evaluate differences in percentages of cells and in the Mean Fluorescence Intensity (MFI) among the groups. A p < 0.05 was considered statistically significant. No power calculation was performed.

## 3 Results

### 3.1 Percentage of cells PKH26 marked and viability

Thymocytes or splenocytes were labeled for PKH26, and up to 95 % cell viability was obtained. Single thymocytes and splenocytes were analyzed, as shown (FSC-A *vs.* FSC-H plot) in Fig. 1A and E, respectively. A classical pattern of size *vs.* granularity was obtained for thymocytes (Fig. 1B) and splenocytes (Fig. 1F). PKH-26 *vs.* granularity dot plots show PKH-26 positive thymocytes (Fig. 1C) and splenocytes (Fig. 1G), up to 84 % of thymocytes were PKH-26+. Around 90 % of splenocytes were marked—Fig. 1D and 1h show autofluorescence and marked thymocytes and splenocytes, respectively. The presence of marked thymocytes (Fig. 2A-D) or splenocytes (Fig. 2E-H) transferred from NP mice to NP mice in the thymus, spleen, blood, and lymph nodes is described.

### 3.2 The percentage of PKH-26+ cells was higher in female than in male mice

**Fig. 3:**
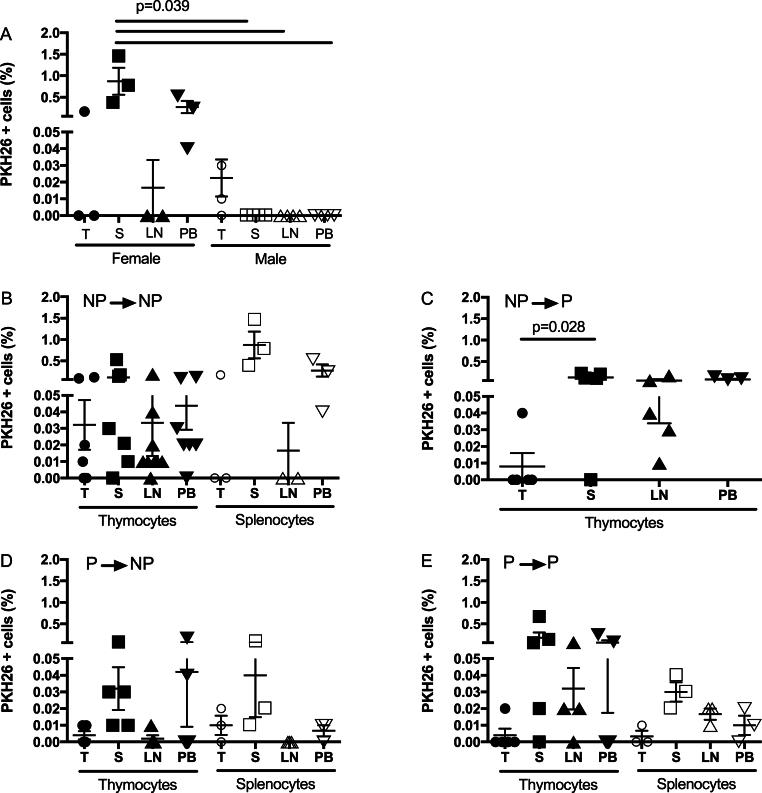
**Percentage of PKH-26+ cells in receptor lymphoid organs and peripheral blood**. Thymocytes or splenocytes were PKH-26 marked as in methods. Five million cells were transferred to the receptor mouse and counted two days later by flow cytometry. A) PKH-26+ thymocytes were counted in lymphoid organs in female and male mice. B-E) PKH-26+ thymocytes or splenocytes were quantified in lymphoid organs in the following groups: **NP→NP, NP→P, P→NP** and **P→P**. Data represented as mean ± SEM. Kruskal-Wallis test, Dunn's multiple comparisons test, IC 95 %. The significance value was p < 0.05. T: thymus; S: spleen; LN: lymph nodes; PB: peripheral blood. Filled symbols: Thymocytes. Open symbols: Splenocytes. Each symbol shows a mouse.

PKH-26+ cells were transferred, and after 48 h, receptor lymphoid organs were collected. Marked cells were quantified by flow cytometry in female and male mice. In Fig. 3A, we analyzed the cell migration dynamics by comparing female and male receptor mice. In most organs, we observed a higher frequency of PKH-26+ cells in female samples than in males (n = 4), except for the male thymus. Also, the percentage of PKH-26+ splenocytes in the female spleen was higher than in the male spleen (p = 0.039), lymph nodes (p = 0.039), and peripheral blood (p = 0.039).

### 3.3 PKH-26+ location to lymphoid tissues in female mice with and without pregnancy

Fig. 1, Fig. 2 show the strategy to label cells with PKH-26 and count those cells in receptor tissues; in Fig. 3, we show the percentage of PKH-26+ cells for each mouse and every condition with NP or P mice. The percentage of PKH-26+ thymocytes and splenocytes from **NP→NP** mice is shown in Fig. 3B. No statistical difference was found between PKH-26+ thymocytes and splenocytes in receptor lymphoid organs. In the case of **NP→P** group (Fig. 3C), we observed a statistical difference between PKH-26+ thymocytes in the spleen vs. in the thymus (p = 0.028), and no statistical difference was noted among the other lymphoid organs. No statistical difference was observed between the proportion of PKH-26+ thymocytes and splenocytes in the **P→NP** and **P→P** groups (Fig. 3 D and E, respectively); also, no difference was described among receptor lymphoid organs. We observed a significant difference between the PKH-26+ thymocytes in lymph nodes for **NP→P *vs.* P→NP**, with a higher frequency of cells in the **NP→P** group (Fig. 3C vs D).

**Fig. 4:**
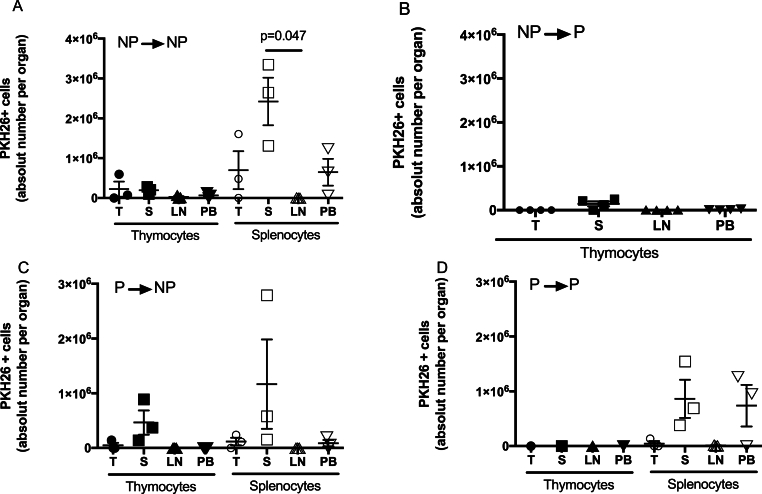
**Absolut number of PKH-26+ cells in receptor lymphoid organs and peripheral blood**. Thymocytes or splenocytes were PKH-26 marked as in methods. Five million cells were transferred to the receptor mouse and quantified after 48 h by flow cytometry. Data represents mean ± SEM. Kruskal-Wallis test, Dunn's multiple comparisons test, IC 95 %. The significance value was p < 0.05. T: thymus; S: spleen; LN: lymph nodes; PB: peripheral blood. Filled symbols: Thymocytes. Open symbols: Splenocytes. Each symbol shows a mouse.

Fig. 4 shows the absolute number of PKH-26+ thymocytes or splenocytes quantified 48 h after the transfer. The highest number of cells were detected in the **NP→NP** condition; however, this was not statistically different from the other groups (**NP→P, P→NP** or **P→P**). In general, the number of PKH-26+ splenocytes detected in the lymphoid organs was higher than that of PKH-26+ thymocytes; however, no statistically significant differences were observed. The number of PKH-26+ splenocytes was higher in the spleen compared to LN in the **NP→NP** group (p = 0.047).

Finally, to explore if PKH-26+ cells express a differential location to lymphoid organs around the moment of birth, PKH-26+ thymocytes or splenocytes were transferred from Pregnant (15–16 DPC) to PostPregnant (PP) mice (1–3 days after birth). A similar frequency of thymocytes and splenocytes in lymphoid organs was observed in the PP receptor mice, compared to NP or P mice (Suppl. Table 1).

## 4 Discussion

Several physiological changes occur during pregnancy; among these changes, an increase in plasma and blood cells has been thoroughly described [20,21]. We asked if, during this physiological leukocytosis, the leukocyte mobility could follow a different migration pattern than in the absence of pregnancy. Using an experimental model, we addressed this problem. We report the proportion of PKH26+ thymocytes and splenocytes located in different lymphoid tissues in the presence or absence of pregnancy.

The traffic of leukocytes among lymphoid organs increases in some phases of pregnancy; it has been reported that the traffic of monocytes toward the uterus is necessary for the correct development of the initial stages of labor by increasing uterine contractility [22]. In physiologic conditions without pregnancy, the classic cell migration pattern goes from primary to secondary lymphoid tissue to non-lymphoid tissue, where they do an effector function [23]. It has been recently determined that primary lymphoid organs are also in the pathway of lymphocyte trafficking [[24], [25], [26]].

First, when we compared the proportion of PKH26+ cells from female mice and transferred to female and male mice, we observed that female mice had a higher proportion of transfer cells in lymphoid tissue than male mice, indicating that the cell migration is more frequent in females than males. Also, it could suggest that our observations in pregnant and non-pregnant mice are valid because males showed a meager number of PKH-26+ cells; in contrast, cells persisted (at least for two days) in females in all the tissues analyzed.

The number of marked cells in a particular tissue depends on the migration rate, proliferation *in situ*, cell death, or outflow from the tissue. Also, pregnancy is a unique condition where change in the cellularity and size of lymphoid organs have been reported. We hypothesized that the cell itself or the pregnancy environment could act to regulate the migration of leukocytes in mice. In agreement with a previous report [14], we observed that the absolute number of cells increased in the spleen of pregnant mice. Also, our results showed that the number of PKH26+ splenocytes is higher in the spleen of pregnant mice; however, we do not know if this is because of higher cell proliferation or a lower outflow of cells from the tissue. More studies are necessary to clarify this question.

Our results showed that the proportion of PKH26+ cells in lymphoid organs was similar between pregnant and non-pregnant conditions, however our study has a limitation because a small number of data. We observed that PKH26+ splenocytes were located mainly in the spleen and, to a lesser extent, in lymph nodes. The percentage of labeled thymocytes in the spleen and lymph nodes was low, indicating that mature resting lymphocytes populate these tissues. Also, in agreement with a previous report [26], the number of PKH-26+ thymocytes detected in the thymus was low in most cases, indicating that thymocytes can return to the thymus. Still, the pregnancy does not alter this pattern. Since the proportion of PKH26+ thymocytes or splenocytes is higher in organs than in the peripheral blood, we believe that the number of PKH26+ cells reflects the proportion of cells in the organ. More analysis must be performed to solve this question. Finally, our results suggest that the mobility of thymocytes and splenocytes in the receptor mice is similar between pregnancy and recently after birth. Also, a percentage of PKH-26+ cells were detected in the placenta of receptor pregnant mice (data not shown), and the number was similar to the lymphoid organs analyzed, suggesting that the placenta could support certain immune vigilance and interchange between mother and fetus. The percentage of microchimerism in humans detected with molecular biology or histologic tests is similar to the percentage of cells that we detected [[27], [28], [29]]; this opens the option to explore aspects of microchimerism in a mice model, doing experiments that are not possible in humans for ethical reasons.

## 5 Conclusions

Splenocytes and thymocytes have similar migration patterns to lymphoid organs irrespective of pregnancy, but female receptors persistently retain transferred cells.

## Ethical considerations

The experiments were performed according to the guidelines for using laboratory animals in the Escuela Nacional de Ciencias Biológicas del Instituto Politécnico Nacional. The study was approved by the Animal Ethics Committee of the Escuela Nacional de Ciencias Biológicas del Instituto Politécnico Nacional, Mexico (ZOO-025-2018).

## Consent for publication

Not applicable.

## Availability of data and materials

The datasets used and analyzed during the current study are available from the corresponding author upon reasonable request.

## Funding

Not applicable.

## CRediT authorship contribution statement

**Gabriela T. Cruz-Cureño:** Writing – original draft, Data curation. **Marina Ch Rosales-Tarteaut:** Writing – original draft, Data curation. **Lourdes A. Arriaga-Pizano:** Supervision, Formal analysis. **Luvia E. Sánchez-Torres:** Supervision, Formal analysis. **Denisse Castro-Eguiluz:** Writing – review & editing, Supervision. **Jessica L. Prieto-Chávez:** Validation, Supervision, Software. **Rodolfo Pastelin-Palacios:** Supervision, Resources. **Ana Flisser:** Validation, Supervision, Resources. **Arturo Cérbulo-Vázquez:** Writing – review & editing, Validation, Supervision, Methodology, Formal analysis, Conceptualization.

## Declaration of competing interest

The authors declare that they have no known competing financial interests or personal relationships that could have appeared to influence the work reported in this paper.

## Data Availability

Data will be made available on request.

## Abbreviations

SLO: Secondary Lymphoid Organs
NP: Non-Pregnant
P: Pregnant
dpc: days post coitus
MFI: Mean Fluorescence Intensity
T: Thymus
S: Spleen
LN: Lymphnode
PB: Peripheral Blood
PP: PostPregnant
