# Supplementary material for: Splenocytes and thymocytes migration patterns between lymphoid organs in pregnancy
Source: Biochem Biophys Rep. 2024 Jul 2;39:101769. doi: 10.1016/j.bbrep.2024.101769 (PMC11268130; doi:10.1016/j.bbrep.2024.101769)
Supplement: Multimedia component 1 [file mmc1.docx]

**Suppl. Table 1. Transfer PKH26+ cells in lymphoid organs or blood in the perinatal period.**

| Donor 🡪 | Thymus | | | | |  | | Spleen | | | |  |
| --- | --- | --- | --- | --- | --- | --- | --- | --- | --- | --- | --- | --- |
| Receptor 🡪 | T  (n=3) | S  (n=3) | LN  (n=3) | PB  (n=3) | *p* | | T  (n=3) | S  (n=3) | LN  (n=3) | PB  (n=3) | *p* | |
| P → NP | 0.003± 0.01 | 0.02± 0.01 | 0 | 0 | ns | | 0.01± 0.01 | 0.04±  0.04 | 0 | 0.01±  0.01 | ns | |
| P → P | 0.007± 0.01 | 0.27± 0.34 | 0.04± 0.02 | 0.08± 0.14 | ns | | 0.003± 0.006 | 0.03± 0.01 | 0.02± 0.01 | 0.01±  0.01 | ns | |
| P → PP | 0.01±  0.01 | 0.12±  0.10 | 0.06±  0.05 | 0.14±0.16 | ns | | ND | ND | ND | ND | -- | |
| *p'* | ns | ns | ns | ns |  | | ns | ns | ns | ns |  | |

Thymus (T), Spleen (S), Lymph node (LN), Peripheral Blood (PB). Data represented as mean±SD. Kruskal-Wallis test, Dunn's multiple comparisons test, IC 95%. The significance value was p<0.05. ns: not statistically significant; ND: not determined.

**Discussion**

Several physiological changes occur during pregnancy; among these changes, an increase in plasma and blood cells has been thoroughly described (20, 21). We asked if, during this physiological leukocytosis, the leukocyte mobility could follow a different migration pattern than in the absence of pregnancy. Using an experimental model, we addressed this problem. We report the proportion of PKH26+ thymocytes and splenocytes located in different lymphoid tissues in the presence or absence of pregnancy.

The traffic of leukocytes among lymphoid organs increases in some phases of pregnancy; it has been reported that the traffic of monocytes toward the uterus is necessary for the correct development of the initial stages of labor by increasing uterine contractility (22). In physiologic conditions without pregnancy, the classic cell migration pattern goes from primary to secondary lymphoid tissue to non-lymphoid tissue, where they do an effector function (23). It has been recently determined that primary lymphoid organs are also in the pathway of lymphocyte trafficking (24, 25, 26).

First, when we compared the proportion of PKH26+ cells from female mice and transferred to female and male mice, we observed that female mice had a higher proportion of transfer cells in lymphoid tissue than male mice, indicating that the cell migration is more frequent in females than males. Also, it could suggest that our observations in pregnant and non-pregnant mice are valid because males showed a meager number of PKH-26+ cells; in contrast, cells persisted (at least for two days) in females in all the tissues analyzed.

The number of marked cells in a particular tissue depends on the migration rate, proliferation *in situ*, cell death, or outflow from the tissue. Also, pregnancy is a unique condition where change in the cellularity and size of lymphoid organs have been reported. We hypothesized that the cell itself or the pregnancy environment could act to regulate the migration of leukocytes in mice. In agreement with a previous report (14), we observed that the absolute number of cells increased in the spleen of pregnant mice. Also, our results showed that the number of PKH26+ splenocytes is higher in the spleen of pregnant mice; however, we do not know if this is because of higher cell proliferation or a lower outflow of cells from the tissue. More studies are necessary to clarify this question.

Our results showed that the proportion of PKH26+ cells in lymphoid organs was similar between pregnant and non-pregnant conditions, however our study has a limitation because a small number of data. We observed that PKH26+ splenocytes were located mainly in the spleen and, to a lesser extent, in lymph nodes. The percentage of labeled thymocytes in the spleen and lymph nodes was low, indicating that mature resting lymphocytes populate these tissues. Also, in agreement with a previous report (26), the number of PKH-26+ thymocytes detected in the thymus was low in most cases, indicating
